# Isolation and Screening of Extracellular PGPR from the Rhizosphere of Tomato Plants after Long-Term Reduced Tillage and Cover Crops

**DOI:** 10.3390/plants9050668

**Published:** 2020-05-25

**Authors:** Maria Chiara Guerrieri, Elisabetta Fanfoni, Andrea Fiorini, Marco Trevisan, Edoardo Puglisi

**Affiliations:** 1 Department for Sustainable Food Process, Università Cattolica del Sacro Cuore, 29122 Piacenza, Italy; mariachiara.guerrieri@unicatt.it (M.C.G.); elisabetta.fanfoni01@icatt.it (E.F.); edoardo.puglisi@unicatt.it (E.P.); 2 Department of Sustainable Crop Production, Università Cattolica del Sacro Cuore, 29122 Piacenza, Italy; andrea.fiorini@unicatt.it

**Keywords:** conservation agriculture, biostimulants, diazotrophs, phosphate solubilizers, IAA production, siderophores, *Sclerotinia sclerotiorum*

## Abstract

Plant growth promoting rhizobacteria provide an innovative solution to address challenges in sustainable agro-ecosystems, improving plant growth as well as acting as agents of biocontrol. In this study autochthonous bacteria were isolated from the rhizosphere of processing tomato plants (*Solanum lycopersicum* L.) cultivated with conservation agriculture practices (i.e., reduced tillage and cover crops), and evaluated for both growth-promoting activities (PGPAs), and antagonistic potential against the phytopathogenic pest *Sclerotinia sclerotiorum*. Considering the several activities of PGPR, we decided to structure the screening with a hierarchic approach, starting from testing the capability of fixing nitrogen. The obtained bacteria were processed through the molecular typing technique rep-PCR (Repetitive Extragenic Palindromic) in order to discriminate microbial strains with the same profiles, and identified via 16S rDNA sequencing. Thirty-eight selected isolates were screened in vitro for different activities related to plant nutrition and plant growth regulation as well as for antifungal traits. Isolated bacteria were found to exhibit different efficiencies in indoleacetic acid production and siderophore production, phosphate solubilization and biocontrol activity against the widespread soil-borne plant pathogen *S. sclerotiorum*. All the 38 bacterial isolates showed at least one property tested. With a view to detect the suitable candidates to be developed as biofertilizers, the selected isolates were ranked by their potential ability to function as PGPR. Thus, consortium of native PGPR bacteria inoculants may represent a suitable solution to address the challenges in sustainable agriculture, to ensure crop yield and quality, lowering the application of chemicals input.

## 1. Introduction

Plants can no longer be seen as standalone entities; it has now been ascertained the role of plant microbiota in promoting plants fitness thanks to an intimacy of interactions between the plant and a wide diversity of microorganisms both inside and outside plant tissues, in the endosphere and rhizosphere respectively [1]. This is especially the case for soils where conservation agriculture practices (i.e., reduced tillage, cover crops) are performed [2,3], due to enhanced soil fertility conditions [4]. Bacteria able to colonize plant root systems and promote plant growth are referred to as plant growth promoting rhizobacteria (PGPR) [5,6]. PGPR exert a number of positive effect to plants, ranging from direct influence mechanisms, aimed at the plant nutrition and plant growth regulation, to indirect effects, related to the biocontrol activity [7,8]. Thus, PGPR appear to promote plant growth by acting both as biofertilizers and biopesticides [9]. In fact, the use of PGPR in agriculture is steadily increasing, and several studies pointed out the rhizobacteria growth promoting activity through the capability to promote alternative nutrient uptake pathway [10], the suppression of disease-causing organisms because of a broad-spectrum antifungal activity [11] and the promotion of beneficial effects on plant growth by the production of phytohormones [12,13]. Beside this, PGPR are also involved in amelioration of abiotic stress such as dryness and salinity stress [14], in revitalization of soil quality and plants growth [15] and enhancement phytoremediation of heavy metals [16].

Despite the considerable number of studies reporting the efficacy of PGPR in several commercial agriculture crops, the application of these PGPR on a regular agricultural management is still underexplored, in comparison to the amount of agricultural land cultivated worldwide [17]. Interest in the beneficial rhizobacteria associated with horticultural crops has extensively increased, and several studies clearly demonstrated the positive and beneficial effects of PGPR on growth and yield of different horticultural crops [18,19]. Tomato (*Solanum lycopersicum* L.) is one of the most widespread horticultural crops worldwide and is ranked second next to potato [20]. World production for processing tomato was estimated at 34,8 million tons in 2018 [21]. In Italy, a total area of 91,406 ha was cultivated with tomatoes in 2019, of which 81.1% is represented by processing tomatoes (74,082 ha) and the remaining is destined to tomato for fresh consumption (ISTAT, 2019) [22].

The rhizosphere of tomato plants is the preferential site for the isolation of effective PGPR with growth plant and defense ability [23]. According to the literature, *Pseudomonas* and *Bacillus* species are the genera most isolated from rhizosphere of tomato plant and well biochemically characterized in vitro and in vivo for their plant growth promoting (PGP) traits [24,25,26,27]. Overall, native rhizospheric *Pseudomonas* and *Bacillus* species are well known by their strong phosphorus solubilization activity, and by their IAA and siderophore production abilities [28,29]. Along with these plant growth promoting properties, PGPR with nitrogen-fixation ability can play a significant role in supplying the nitrogen requirement for plant growth and yield production. Other researchers screened tomato rhizosphere for detecting effective diazotrophic bacteria, for their application to integrated crop cultivation practices [30]. Furthermore, tomato production has shown limitations arising from the use of cultivars susceptible to disease especially to Sclerotinia Stem Rot induced by the widespread soil-borne plant pathogen *Sclerotinia sclerotiorum* [31]. Biocontrol activity against *S. sclerotiorum* is widely reported for *Pseudomonas* and *Bacillus* strains [32,33,34]. Moreover, strains isolated from tomato rhizosphere and belonging to genera other than *Pseudomonas* and *Bacillus* are under-explored for plant-growth promotion and defense.

In general, the bacterial diversity detected in the rhizosphere is represented mainly by species belonging to three dominant phyla such as Proteobacteria, Firmicutes, and Actinobacteria [35], where the most common genera reported include *Bacillus*, *Pseudomonas*, *Enterobacter*, *Erwinia*, *Serratia*, *Arthrobacter*, *Rhizobium*, *Agrobacterium*, *Burkholderia*, *Azospirillum*, *Azotobacter*, *Mycobacterium*, *Flavobacterium,* and *Micrococcus* [36]. However, a shortage of studies is referred to the microbial diversity that is potential induced by reduced tillage (RT) and cover crops (CCs) on field. Indeed, while it is now well known that CCs increase the biomass of a microbial community [37,38], there is still a paucity of information on the effect of long-term RT management on soil microbial structure and diversity, rather than a conventional tillage (CT) management based on soil plowing [37,39].

The objective of this study was to isolate and characterize beneficial bacteria that are present in processing tomato (*Solanum lycopersicum*) rhizosphere after a long-term RT soil management. We focused on the identification of potential PGPR isolates by using selective or semi-selective culture media, by characterizing their specify metabolic features, by screening them with rep-PCR and by determining the identities of selected bacteria using *16S rRNA* gene sequencing. Our approach was to assess the plant growth promoting activities (PGPAs) of the isolates of *Solanum lycopersicum* rhizosphere soil by conducting in vitro qualitative and quantitative assays for traits related to (i) mineral nutrition, including phosphate (*P*) solubilization, nitrogen (*N*) fixation and siderophores production, (ii) the production of growth hormones like indole acetic acid (IAA), and (iii) antagonism against fungal pathogen such as *Sclerotinia sclerotiorum*. Finally, isolates were ranked by their potential ability to function as PGPR.

## 2. Material and Methods

### 2.1. Field Site and Samples Collection

Soil sampling occurred in July 2019 in a commercial field situated in Gabbioneta-Binanuova (45°12’03.0” N 10°12’27.8” E), Cremona, Po Valley (Northern Italy). At the selected field site conservation agriculture practices have been adopted since 2011. Specifically: (i) A mixture of cover crops (CCs), composed by rye (*Secale cereale* L.), hairy vetch (*Vicia villosa* Roth), and radish (*Raphanus sativus* L.), was sown each year after harvesting the previous main crop and terminated right before the next seedbed preparation; (ii) reduced-tillage (RT) operations, which consisted of a ripper passage (25 cm depth) and one/two spring harrowing (10–15 cm depth), were annually performed before planting the main crop. The crop sequence was a 3-year crop rotation including maize (*Zea mays* L.), soybean (*Glycine max* (L.) Merr.), and processing tomato (*Solanum lycopersicum* L.).

Soil samples adhered to the roots of tomato plants were manually separated from the surrounding bulk soil and collected. The plants were carefully uprooted from soil, collected in sterile polybags and stored at 4 °C for the isolation of rhizobacteria. The rhizosphere and rhizoplane soil were separated from the bulk soil following the method proposed by Barillot et al. [40]. Briefly, bulk soil was removed shaking plants by hand for 10 min vigorously, paying attention to the roots’ integrity, as long as the roots’ non-adhering soil particles were completely removed. In order to collect rhizosphere and rhizoplane soil, the root system was washed with 500 mL of 0.9% NaCl added with Tween 80 (0.01% *v/v*) and afterwards 150 mL of bacterial suspension were incubated at 25 °C for 90 min with shaking at 180 rpm.

### 2.2. Isolation of Putative Diazotrophic Vacteria

Considering the many activities of PGPR, it has been decided to start the screening analysis of bacteria that may fix atmospheric nitrogen using two different N-free semi-solid media, NFb (New Fábio Pedrosa) and LGI (Liquid Glucose Ivo) [41] and comparing the potential growth with the bacterial strain Azospirillum brasilense Sp7 (DSM 1690), used as positive control [42]. The isolation of the putative diazotrophic bacteria was carried out following the method proposed by Ambrosini and Passaglia [43]. After incubation, each suspension was diluted in a sterile NaCl 0.9% solution until reaching 10−3, in triplicates. One hundred μL of the initial suspension (100) and serial 3-fold dilutions (10−1, 10−2, 10−3) were inoculated in 4 mL of NFb and LGI, in duplicate, and incubated for 4–7 d at 30 °C until the growth of a veil-like pellicle near the surface of the culture medium. The colour change of the medium from green-blueish to blue for NFb and from green to yellow for LGI, was another indicator for the bacterial growth. Pellicles were re-inoculated in new vials under the previous conditions in order to reduce the number of scavenger bacteria. Usually, from each vial only one bacteria isolate will be obtained, thus biofilms were streaked in LB agar (Luria-Bertani) (Oxoid, Basingstoke, UK) to confirm this and bacterial cultures were incubated at 30 °C for 15–24 h under aerobic conditions. After incubation, a morphological visual screening was performed, and a first selection was made to exclude the same isolates.

### 2.3. Rep-PCR

The genetic diversity among the isolates obtained was assessed by means of rep-PCR genotyping analysis, in order to discriminate same microbial strains profile [44]. DNA extraction from bacterial isolates was performed using the Microlysis Plus kit (Microzone, Haywards Heath, UK). Repetitive extragenic palindromic PCR was performed using GTG5 (5′-GTGGTGGTGGTGGTG-3′) as a primer [45]. PCR amplification was carried out in a 25 μL reaction mixture containing 1 μL DNA sample, 0.5 μL of GTG5 primer (100 μM), and 23.5 μL Megamix (Microzone). PCR was performed in a T100™ Thermal Cycler (Bio-Rad Laboratories, Hercules, CA, USA) with the following steps: Initial denaturation at 95 °C for 7 min, followed by 30 cycles of denaturation at 90 °C for 30 s, annealing at 40 °C for 1 min, extension at 65 °C for 8 min and a final extension at 65 °C for 16 min. The PCR products were electrophoresed in a 2.5% agarose gel in 1× Tris-acetate-EDTA (TAE) buffer solution at 80V for 2 h and the profiles were visualized with the software Image Lab (Bio-Rad). The comparative analysis of the resulting fingerprints was performed using the software Geljv.2.0 [46].

### 2.4. Taxonomic Identification of Unique Isolates

The taxonomical identification of the bacterial isolates was carried out by amplification and sequencing of the 16S rRNA using the primers P1 (5′-GCGGCGTGCCTAATACATGC-3′) and P6 (5′-CTACGGCTACCTTGTTACGA-3′) [47]. PCR amplification was carried out in a 25 μL reaction mixture containing 2 μL DNA sample, 0.5 μL of each primer (50 μM), 22 μL Megamix (Microzone). PCR was performed in a T100™ Thermal Cycler (Bio-Rad) with the following steps: Initial denaturation at 95 °C for 5 min, followed by 30 cycles of denaturation at 92 °C for 1 min, annealing at 55 °C for 1 min, extension at 72 °C for 1 min and a final extension at 72 °C for 10 min. PCR products were run on 1% agarose gel in 1× Tris-acetate-EDTA (TAE) buffer solution at 100V for 60 min and the profiles were visualized with the software Image Lab (Bio-Rad). After the DNA purification by NucleoSpin®Gel and PCR clean-up (MACHEREY-NAGEL, Duren, Germany) and quantification by electrophoresis using 1% agarose gel and Marker II (Roche diagnostics GmbH, Mannheim, Germany), the amplified fragments were subjected to Sanger sequence analysis. Sanger sequencing of PCR products was carried out at GATC Biotech (Ebersberg, Germany). The 16S ribosomal DNA sequences were then analysed through the NCBI-BLAST server. The BLAST result revealed the identity of the query sequences based on their percentage query coverages and sequence identities. Bacterial isolates with human and plant pathogenic properties were identified on the basis of literature info and discarded.

### 2.5. Phosphate Solubilization Ability Assessment

All bacterial isolates were screened for phosphate solubilization on the GY/Tricalcium phosphate medium [43] containing tricalcium phosphate (Ca_3_PO_4_)_2_ as insoluble source of phosphorus. Each bacterial isolate was spot inoculated onto these plates and incubated at 30 °C for seven days. Plates were observed for development of a halo zone around the colony, which is indicative of tricalcium phosphate solubilization. This phosphate solubilization ability was analyzed measuring the halo’s diameter according the method proposed by Ambrosini and Passaglia [43]: isolates without a halo (halo = 0 cm) were considered as non-phosphate solubilizers or level 1 phosphate solubilizers, isolates with a halo bigger than 0 cm up to 1 cm were considered level 2 phosphate solubilizers, and isolates with a halo bigger than 1 cm were considered level 3 phosphate solubilizers.

### 2.6. Determination of the Indole Acetic Acid Production

IAA production by bacterial strains was estimated using the Salkowski reagent, which consisted of 12 g of FeCl_3_ per L in 7.9 M H_2_SO_4_ [48]. Briefly, 1.5 mL of each bacterial cultures (24 h old) were centrifugated at 5000–8000 rpm for 10 min, supernatant was discarded and the pellet was resuspended in the same amount of sterile distilled water. The concentration of bacterial cultures was adjusted to an O.D_600_ = 0.1. An aliquot of 10 μL from each culture was inoculated both in 5 mL of LB Broth supplemented with 500 μL DL-Tryptophan (0.01%) and as well in LB broth without DL-tryptophan and then incubated at 30 °C for 72 h, with shaking at 180 rpm. After incubation, 1000 μL of bacterial cultures were centrifugated at 6000 rpm for 10 min. Five hundred microliters of Salkowski reagent was added to 1 mL of collected supernatant and after 30 min incubation in dark the reddish color developed which indicated the IAA production. To quantify IAA, absorbance was taken at 540 nm by using UV/visible spectrophotometer. The IAA concentration was estimated with a standard curve of IAA.

### 2.7. Siderophores Production Assay

Quantitative estimation of siderophores production was done using CAS (Chrome Azurol Sulphonat) reagent [49]. This test was carried out following the protocol proposed by Arora and Verma [50], the modified microplate (96 wells plate) method was performed. Briefly, bacterial cultures (48 h old) were centrifuged at 10,000 rpm for 10 min, cell pellets were discarded, and supernatant was used to estimate siderophores. Supernatant (100 μL) of each bacterial culture was added in separate wells of microplate followed by the addition of 100 μL CAS reagent. After 20 min optical density was taken at 620 nm using microplate reader. Siderophore produced by strains was measured in percent siderophore unit (psu) which was calculated according to the following formula:

(1) $$Siderophore\;production=\frac{\left(A_{r}-A_{s}\right)\times 100}{A_{r}}$$

where *A*_*r*_ = absorbance of reference (CAS solution and uninoculated broth), and *A*_*s*_ = absorbance of sample (CAS solution and cell-free supernatant of sample). The chelating agent EDTA (Ethylenediaminetetraacetic acid) was used as positive control.

### 2.8. In Vitro Assessment of Antifungal Activity

Bacterial isolates were screened for antifungal activities against *Sclerotinia sclerotiorum* (DSM 1946) using dual culture assay [51] on a potato dextrose agar (PDA) medium. A mycelial agar disc of 5 mm diameter of the pathogen was taken from an actively grown PDA culture of fully grown 7-day-old culture and was placed on one side of the PDA plates about 3 cm from the edge. A loopful of each antagonistic bacterial isolate, from an overnight culture, was streaked 3 cm away from mycelia disc. Only the culture of the fungal pathogen was inoculated in control plate. After 5–7 days of incubation at 25 °C, the antagonistic activity was observed by measuring the size of the growth inhibition zone and the percentage of growth inhibition (PGI) was calculated using the formula:

(2) $$PGI\;\left(\%\right)=\frac{KR-R1}{KR}\times 100$$

where *KR* represents the colony diameter of the pathogen in the control plate, and *R1* represents the colony diameter in the treated plate.

### 2.9. Data Analysis

Results were expressed as means + standard errors (SE). For the antifungal activity analysis differences between means were determined by one-way ANOVA with post-hoc Tukey’s HSD (honestly significant difference) test with the level of significance established at *p* < 0.05.

In order to select the most performing bacterial isolates, the strains were ranked for their potential to promote plant growth and defense ability. The ranking covers all the data obtained from the in vitro assays. Briefly, the ranking was made assigning to each isolate a value in a range from zero to one to each PGP and antifungal traits. For the biocontrol activity, IAA and siderophore production, the maximum value for each evaluated trait was converted to the 100%, then, all the other values were proportioned to it. For phosphate solubilization, to each of four solubilization level identified, a value was assigned in a ratio scale of 0.25, that is, level 1 = 0.25, level 2 = 0.50, level 2 + = 0.75 and level 3 = 1. While for the nitrogen fixation trait, only two output were obtained from the assay, that is the positive (+) or the negative (−) growth, hence only the two value 0 or 1 were appointed. For every single bacterial strain all the values obtained for each PGPR traits tested were added up. Furthermore, the ranking obtained was compared with the bonitur scale, an arbitrary ranking commonly used to assess the bacteria isolates with high plant growth promotion potential [30,52].

## 3. Results

### 3.1. Isolation, Putative Diazotrophic Bacteria Identification, Rep-PCR, and Molecular Characterization of Rhizobacteria

**Figure 1:**
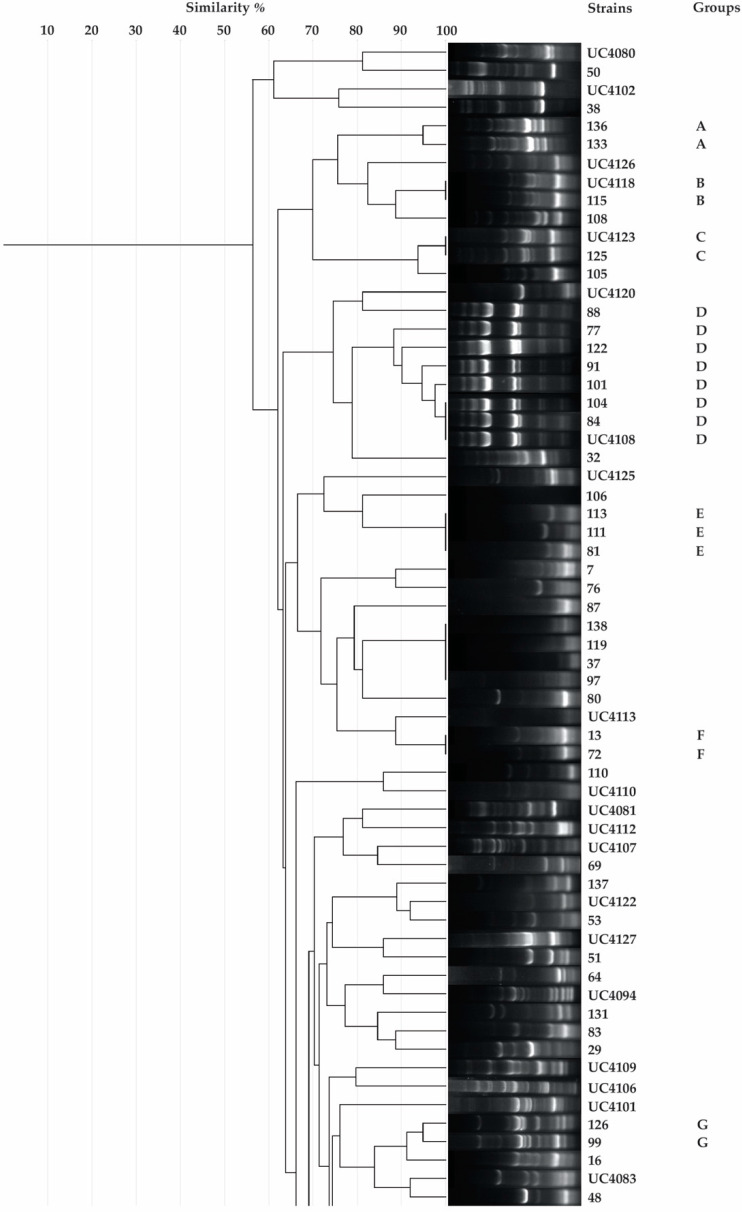

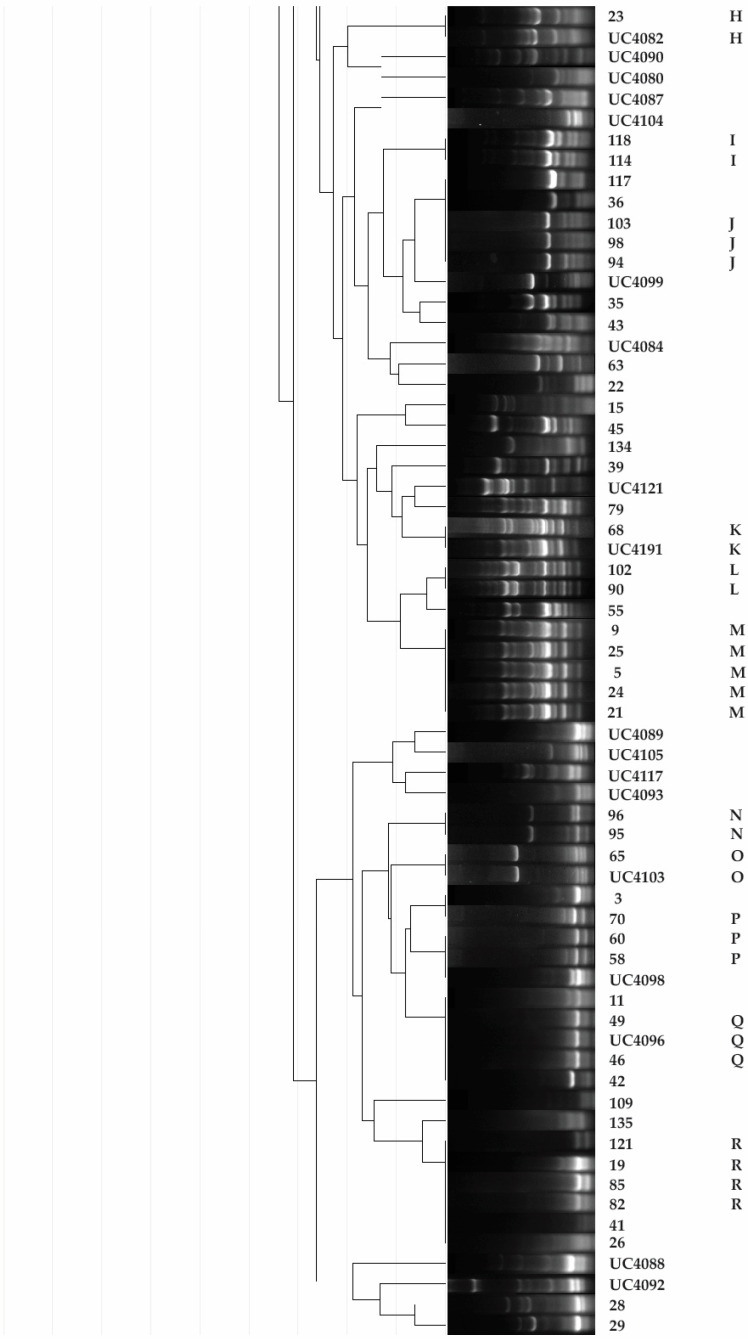
Dendrogram analysis of rep-PCR (Repetitive Extragenic Palindromic) fingerprints performed using the software Geljv.2.0. Same bacterial strains belong to the same group.

According to the protocol proposed by Ambrosini and Passaglia [43], from each vial only one bacteria isolate is obtained. However, in our study, streaking the veil-like pellicle developed in NFb semi-solid medium on LB agar plates, from some single vials more than one isolate were obtained. Hence, a total of 129 bacterial isolates were discerned from the rhizosphere and rhizoplane soil of tomato plant *Solanum lycopersicum*. Rep-PCR analysis of the all 129 isolates was performed in order to reduce genetic redundancy, and the clustering scheme was constructed by the software Geljv.2.0 (Figure 1). In accordance with the dendrogram of genetic similarity using Dice similarity coefficient index only 34 isolates had similar repetitive-element PCR genomic fingerprint to others isolates, indicating the high diversity in the system. Hence, the bacterial isolates were reduced from 129 to 95. Molecular identification of resulting bacterial strains was done by 16S rRNA gene sequencing analysis. The sequences obtained were submitted to NCBI GenBank using BLAST (Basic Local Alignment Tool). Based on scientific literature we discarded bacterial isolates with the highest possibilities to have human and plant pathogenic properties. The most common human pathogens found were *Stenotrophomonas maltophilia*, *Klebsiella pneumoniae,* and *Klebsiella variicola*. After this literature research the number of bacterial isolates was further decreased from 95 to 38. The BLASTn phylogenetic analyses revealed that these 38 isolates belonged to 9 different genera. Out of these 38 isolates, 10 (26.3%) isolates belonged to genus *Pseudomonas*, 7 (18.4%) were from genus *Stenotrophomonas*, 5 (13.2%) from genus *Klebsiella*, 4 (10.5%) each from genus *Chryseobacterium* and *Enterobacter*, 3 (7.9%) each from genus *Sphingobacterium* and *Kosakonia*, and 1 (2.6%) isolate each belonged to genera *Aeromonas* and *Delftia*. All the bacterial isolates obtained, excluding the pathogenic strains, are reported in Table 1. Since more than one bacterial isolate was obtained from each vial containing the *N*-free semi solid medium NFb and LGI, the 38 isolates screened were re-inoculated in new vials with NFb and LGI medium under the previous condition in order to confirm their potential ability to fix nitrogen. Among the 38 screened isolates, a total of 29 bacteria were confirmed to be putative diazotrophic bacteria, in which *Pseudomonas* was the most abundant nitrogen fixer in tomato rhizosphere and rhizoplane soil (Table 2). Not wanting to focus only on the potential to fix nitrogen, the whole 38 bacteria isolates were assessed for the others potential PGPAs, such as: Tricalcium phosphate solubilization, IAA production, siderophore production, and antifungal activity. 

### 3.2. Qualitative Estimation of Phosphate Solubilization Ability

Among the 38 selected isolates, 18 bacterial strains are able to solubilize phosphate by producing clear zone around the colonies after 7 days of incubation. Two different strains of *Pseudomonas taiwanensis* (isolates UC4117 and UC4122) showed highest phosphate solubilization level (level 2 +). Both isolates showed a significant clear zone around the colonies, wider than the other level 2 bacteria, and close to 1 cm in diameter, hence their ability of phosphate solubilization was indicated as level 2 +. During this assay no level 3 phosphorus solubilizers were obtained, while 11 strains were not able to grow on GY/Tricalcium phosphate medium. Results for the phosphate solubilization assay are shown in Table 2.

### 3.3. Determination of the Indole Acetic Acid Production

To screen for indole acetic acid production the Salkowski reagent was used, which gave different degrees of red to the solution according to the different levels of IAA produced. Qualitative and quantitative analysis of culture supernatant of selected strains isolated revealed production of variable amount of IAA both in the absence and presence of tryptophan (0.01%). The concentration of IAA produced by the rhizobacteria showed variations between 0 and 32.99 μg/mL in presence of L-tryptophan, *Enterobacter tabaci* isolate UC4109, produced maximum IAA (32.99 μg/mL), followed by the isolates UC4086, *Klebsiella oxytoca,* (17.85 μg/mL) and the isolate UC4094, *Enterobacter tabaci*, (15.33 μg/mL). An important variation in the concentration of IAA produced was observed between 0.05 and 33.07 μg/mL even without the IAA precursor tryptophan, in which the isolates UC4098 (*Stenotrophomonas rhizophila*) produced the highest level of IAA (33.07 μg/mL). Quantitative analysis for the indole acetic acid production are shown in Table 2.

### 3.4. Siderophores Production Assay

The quantitative microplate method for siderophore estimation revealed that the concentration of siderophore produced by bacterial isolates varied from 0 to 52.28 psu. *Sphingobacterium canadense*, (isolate UC4107), produced maximum amount of siderophore (52.28 psu) followed by *Stenotrophomonas pictorum*, (isolate UC4089) > *Chryseobacterium ureilyticum*, (isolate UC4102), > *Chryseobacterium rhizosphaerae*, (isolate UC4120) > *Stenotrophomonas pictorum*, (isolate UC4093) > *Chryseobacterium oranimense*, (isolate UC4081). The concentration of siderophore produced by these bacterial strains varied from 51.36 to 47.66 psu. Quantitative analysis results for the siderophore production are shown in Table 3.

### 3.5. In Vitro Assessment of Antifungal Activity

Antifungal activity of the bacterial isolates was assayed against *Sclerotinia sclerotiorum* (DSM 1946) using PDA (Potato dextrose agar) media. Results of dual culture assay showed that all strains have different efficiencies in the inhibition of the mycelial growth (Table 3). On the basis on the in vitro dual culture experiment, the antifungal activity of all bacterial isolates checked varied with percent of growth inhibition (PGI), from 5.23% to 48.02%. Isolate UC4127 (*Klebsiella oxytoca*) showed the strongest antagonisms against the pathogen with the highest PGI value (48.02%), followed by the isolate UC4103 (*Pseudomonas hibiscicola*) with a PGI of 45.10% and the isolate UC4123 (*Klebsiella oxytoca*) with a PGI of 44.57%.

### 3.6. Ranking of Different Plant Growth Promoting Traits

With a view to organize the selected bacterial isolated into a hierarchy, a nonarbitrary ranking approach was elaborated. Each PGPR isolate was ranked on the basis of its in vitro PGP (Plant Growth Promoting) and antifungal assay, considering a range from 0 to 1 for each assayed property. According to the ranking scale the first three positions were obtained by isolate UC4094 (*Enterobacter tabaci*), isolate UC4098 (*Stenotrophomonas rhizophila*), and the isolate UC4109 (*Enterobacter tabaci*). A complete list of ranked strains is shown in Table 4. Furthermore, the collection of bacterial strains was also ranked with the arbitrary bonitur scale approach (data not shown). Same results were obtained for the bacteria with the highest score.

## 4. Discussion

In the current study, 38 promising rhizobacterial isolates were selected out of 129 isolates on the basis of their genetic diversity and in vitro PGP and antifungal assay. These potential PGPR were isolated from rhizosphere soil of tomato plants (*Solanum lycopersicum* L.) after a long-term RT plus CCs soil management, and they were screened in vitro for the different PGP traits, such as nitrogen fixation, phosphate solubilization, IAA and siderophore production, and also for antagonistic potential against the phytopathogenic pest *S. sclerotiorum*.

Isolation of putative PGPR from rhizosphere environment usually results in a large number of isolates [53,54]. Hence, we established a strategy to sort out culturable bacteria strains and restrict them to those highly suspected to have PGP and antifungal traits. We decided to follow a hierarchic approach, starting from testing the capability of fixing nitrogen. Nitrogen is an essential element in plant growth. Hence, the biological nitrogen fixation (BNF) can be considered as one of the major mechanisms by which plants can benefit from microorganisms [55]. Moreover, according to Islam et al. [56] inoculation to tomato plants, under gnotobiotic conditions, with some nitrogen-fixing bacterial strains belonging to phyla Proteobacteria and Firmicutes, had significantly impacted on a variety of growth parameters, such as root and shoot length, seedling vigor and dry biomass. Among the 38 screened isolates, after a re-inoculation in *N*-free semi solid medium, a total of 29 bacteria were confirmed to be diazotrophic bacteria. These data confirmed the occurrence of effective nitrogen-scavenging bacteria in the rhizosphere of tomato plant, and the close association with diazotrophic strains [43,57]. In our study the higher isolation frequency of putative diazotrophic bacteria was for the genus *Pseudomonas*.

Isolates were screened at strains level by using rep-PCR genotyping analysis and then the phylogenetic affiliations were determined. It was necessary to carry out a literature research in order to discard bacterial isolates which have characterizing human and plant pathogenic properties. The BLASTn similarity searches revealed that the most common human pathogens found were *Stenotrophomonas maltophilia* and *Klebsiella pneumoniae,* both global opportunistic pathogen increasingly resistant to multiple antimicrobial agents and responsible for the emergent incidence of nosocomial infections, mainly in debilitated and immunosuppressed individuals [58,59], and *Klebsiella variicola*, a versatile bacterium capable of colonizing different hosts such as plants, humans, insects and animals but currently recognized as a cause of several human infections [60]. The phylogenetic analyses, of the non-pathogenic strains, revealed that 38 isolates belonged to 9 different genera, among which the genus *Pseudomonas* was the most abundant; however, strains belonging to the *Bacillus* genus were not identified, possibly even because the phylum Firmicutes, as a whole, tend to be less abundant in the rhizosphere than other phyla [61]. At phylum level our strains can be divided into only two different phyla, such as Proteobacteria and Bacteroidetes, with the full dominance of Proteobacteria.

A metagenomic approach should be performed in order to be able to provide conclusions and results about microbial diversity of our selected environment; however, previous metagenomic investigation about biodiversity in reduce tillage (RT) with CCs compared with in conventional tillage (CT) practice added value to our results. Legrand et al. [62] discovered that phyla Proteobacteria and Bacteroidetes were more abundant in soil under RT, while the presence of Firmicutes strains was higher under CT. Other studies showed that the highest bacterial richness of prokaryotes is typically found in the top soil layer under RT [39], while at deeper layer the richness of phyla under RT is usually lower than under CT [63]. Moreover, a meta-analysis research carried out on the impact of crop rotation by Venter et al. [64] highlighted how longer study trials produced larger increases in microbial richness, although the opposite was true for microbial diversity; nevertheless the addition of legumes to rotation had no consistent effects on microbial diversity or richness. In addition, Buyer et al. [65] pointed out how vetch CC may increase the amount of Gram-negative bacteria in the rhizosphere of tomato plants.

Hariprasad and Niranjana [66] screened the rhizosphere of tomato plants in order to specifically detect phosphate solubilizing rhizobacteria. Phosphate solubilizing microorganisms (PSM), including bacteria, have an important role in plant growth, making the unavailable insoluble sources of *P* available to the actively growing plants. [67]. Generally, strains belonging to the genera *Pseudomonas* are among the most powerful phosphate solubilizers [68]. In this study 18 isolates out of 38 showed phosphate solubilization, where two different strains of *P. taiwanensis* showed the highest level (Table 2). *Pseudomonas taiwanensis* is a novel species bacterium isolated in 2010 [69], Volmer et al. [70] utilized this bacterium as a biocatalyst by considering its capacity as an organic solvent tolerant, showing its potential for use in contaminated sites. According to the literature, only a few studies report its ability to solubilize phosphate [71,72].

Bacterial IAA producers (BIPs), by input of IAA into the plant’s auxin pool, can have a positive effect on root system elongation and development, thereby helping water and nutrient uptake [73]. Our isolates, with a range between 0 and 32.99 μg/mL of IAA production in presence of the precursor L-tryptophan, produce lower IAA concentration as compared to previous reports [74,75]. *Enterobacter tabaci*, produce maximum IAA (32.99 μg/mL) level. In previous investigation, a strain of *E. tabaci* was isolated from the fruits of tomato plant for its ability to produce the polygalacturonase enzyme, an enzyme that aids in microbial spoilage of fruits and vegetables, with the aim to produce important enzymes in food, drinks, and pharmaceutical industries at affordable prices [76]. So, this property should be taken into account and evaluated before its use as an inoculum in agriculture. In the absent of L-tryptophan, generally a reduction or even a lack of production of IAA was observed [77,78]. However, in the current study, a concentration of IAA with the value of 33.07 μg/mL was achieved for *S. rhizophila* (Table 2). A previous study confirmed the capability of *S. rhizophila* to product high level of IAA also without the precursor L-tryptophan [75].

The siderophores production ability can be classifies either as a direct mechanism or an indirect mechanism of plant growth promoting rhizobacteria. They are organic compound with low molecular masses, produced by microorganisms in order to provide plants with Fe nutrition to enhance their growth under low iron conditions. At the same time, siderophore produced by the PGPR bind the iron in order to reduce the Fe availability and efficiently prevent the propagation of fungal pathogens [79]. In the present study (Table 3) the isolates selected produce higher siderophores concentration, within a range of 0 to 52.28 psu, compare to other reports [52,80]. *Sphingobacterium canadense*, showed the higher level of siderophores production. This is in line with another investigation which indicates some strains of *Sphingobacterium* spp. as greater siderophore-producing bacteria [81]. However, in particular for *S. canadense*, according to the literature, there is no information regarding this microorganism, except scientific proof of its isolation from corn roots [82].

Beside stimulating plant growth by direct mechanisms, PGPR isolated were also screened for their suppressive effects against *S. sclerotiorum*, a widespread soilborne plant pathogen affecting yield and product quality of more than 400 plant species, among which there is tomato [83]. In the current study *Klebsiella oxytoca* showed higher antagonistic activity against the pathogen, with a percent growth inhibition value of 48.01%. *Klebsiella* is a borderline genus, known for its pathogenic properties as well as for their potential in agriculture, indeed *K. pneumoniae* and *K. oxytoca* are able to fix atmospheric nitrogen [84]. The PGP properties of *K. oxytoca* were highlighted in several report [85,86], as well for its capacity to induced systemic resistance (ISR) against soft-rot disease pathogen in tobacco [87]. In general, according to the literature there is a lack of information concerning the antagonistic potential of *K. oxytoca* against the phytopathogenic pest *S. sclerotiorum*.

In order to evaluate the potential PGPR isolates to be possibly commercialized as biofertilizers, biopesticide or biostimulant, our first decision-making approach was to rank the selected isolated bacteria. The PGPR were ranked on the basis of their in vitro PGP and antifungal assayed property. According to the ranking scale the first three positions were obtained by isolates UC4094, UC4098, and UC4109 (Table 4). It is noteworthy to consider that ranking approach is based on the sum of all the assayed property: This is means that every rhizobacteria isolate is evaluated on its capability to express more than one PGP or antifungal property. In the current study, all the 38 bacterial isolates showed at least one property tested. Observing the results achieved by the ranking, with the exception of the isolate UC4109, the other two isolated at the top of the scale, are able to exert all the properties tested but these are expressed at lower levels compared to others that express maybe only one property but at a higher level. So, the use of this index should only help in the thinking of the decision-making approach, and its output should not be considered in the strongest term. Moreover, the use of microbial consortia in the form of bio-product for reduction in the application of chemical fertilizers, pesticides, and related agrochemicals, without compromising the plant yield is currently a significant research area in the field of agriculture [88]. From the perspective of developing a bacterial consortium we can observe that in the current study 38 bacterial isolates with at least one plant growth promoting property were isolated from the rhizosphere and rhizoplane of *Solanum lycopersicum* of a local farm; efforts aimed to discovering indigenous microorganisms that can improve crop development and growth are therefore promising. Consortium of native PGPR bacteria inoculants can indeed have the potential to alleviate challenges of local chemical fertilizers production. Further research will be necessary in order to evaluate the effective PGPR behavior of selected isolates, indeed greenhouse and field trials will be performed. Before the in vivo assay, genome analyses will be needed in order to confirm the absence of pathogenic genes and the presence of potential genes involved in PGPR activities.

## Figures and Tables

**Table 1:** Molecular identification of the bacterial isolates based on 16S rDNA gene sequencing.

Code ^a^	Blast Match						
	Identity	Query Lenght	Bit-Score	Query Cover (%)	E-Value	Ident (%)	Accession No. ^b^
UC4080	*Sphingobacterium detergens*	816	1485	100	0.0	99.51	MT435019
UC4081	*Chryseobacterium oranimense*	1073	1940	99	0.0	99.35	MT435020
UC4082	*Pseudomonas pseudoalcaligenes*	1041	1855	99	0.0	99.13	MT435021
UC4083	*Stenotrophomonas acidaminiphila*	1182	2132	100	0.0	99.24	MT435022
UC4084	*Kosakonia radicincintans*	1123	2061	99	0.0	99.82	MT435023
UC4086	*Klebsiella oxytoca*	1155	2093	99	0.0	99.56	MT435024
UC4087	*Pseudomonas indoloxydans*	1204	2176	99	0.0	99.33	MT435025
UC4088	*Pseudomonas indoloxydans*	1243	2268	100	0.0	99.60	MT435026
UC4089	*Stenotrophomonas pictorum*	1136	2076	100	0.0	99.65	MT435027
UC4090	*Aeromonas caviae*	800	1458	99	0.0	99.87	MT435028
UC4091	*Pseudomonas pseudoalcaligenes*	1178	2109	100	0.0	98.98	MT435029
UC4092	*Kosakonia radicincitans*	1068	1954	100	0.0	99.72	MT435030
UC4093	*Stenotrophomonas pictorum*	1184	2145	99	0.0	99.58	MT435031
UC4094	*Enterobacter tabaci*	1102	2021	99	0.0	99.82	MT435032
UC4096	*Stenotrophomonas pavanii*	996	1808	100	0.0	99.40	MT435033
UC4098	*Stenotrophomonas rhizophila*	990	1810	99	0.0	99.70	MT435034
UC4099	*Enterobacter tabaci*	1075	1965	99	0.0	99.81	MT435035
UC4101	*Klebsiella grimontii*	1056	1914	99	0.0	99.52	MT435036
UC4102	*Chryseobacterium ureilyticum*	1143	2025	99	0.0	98.77	MT435037
UC4103	*[Pseudomonas] hibiscicola*	1159	2080	99	0.0	99.30	MT435038
UC4104	*Stenotrophomonas rhizophila*	1023	1873	100	0.0	99.71	MT435039
UC4105	*Stenotrophomonas pictorum*	1188	2128	99	0.0	99.32	MT435040
UC4106	*Enterobacter ludwigii*	1155	2115	99	0.0	99.91	MT435041
UC4107	*Sphingobacterium canadense*	1152	2080	99	0.0	99.13	MT435042
UC4108	*Chryseobacterium rhizosphaerae*	1162	2117	99	0.0	99.83	MT435043
UC4109	*Enterobacter tabaci*	1164	2102	99	0.0	99.48	MT435044
UC4110	*Kosakonia oryzendophytica*	1150	2045	98	0.0	99.12	MT435045
UC4112	*Pseudomonas taiwanensis*	1036	1890	99	0.0	99.81	MT435046
UC4113	*[Pseudomonas] hibiscicola*	1251	2244	99	0.0	99.04	MT435047
UC4117	*Pseudomonas taiwanensis*	1066	1949	99	0.0	99.81	MT435048
UC4118	*Klebsiella oxytoca*	1185	2158	99	0.0	99.66	MT435049
UC4120	*Chryseobacterium rhizosphaerae*	1212	2215	99	0.0	99.75	MT435050
UC4121	*Sphingobacterium siyangense*	985	1725	99	0.0	98.28	MT435051
UC4122	*Pseudomonas taiwanensis*	1162	2102	99	0.0	99.31	MT435052
UC4123	*Klebsiella oxytoca*	1150	2084	99	0.0	99.56	MT435053
UC4125	*Delftia tsuruhatensis*	1022	1869	99	0.0	99.90	MT435054
UC4126	*Pseudomonas japonica*	1151	2087	99	0.0	99.31	MT435055
UC4127	*Klebsiella oxytoca*	1187	2165	99	0.0	99.83	MT435056

^a^ Code for the selected strains with best PGP traits. ^b^ GeneBank sequence accession numbers of selected strains.

**Table 2:** Plant growth promotion traits of the rhizobacteria.

Code	Identity	*N* Fixation	*P* Solubilization	IAA Production (μg/mL)			
		Growth on Nfb		w/Try		w/o Try	
				s.e.m	Tukey	s.e.m	Tukey
UC4080	*Sphingobacterium detergens*	−	−	1.42 ± 1.11	ab	0.39 ± 0.14	b
UC4081	*Chryseobacterium oranimense*	−	Level 1	1.34 ± 0.01	ab	0.86 ± 0.02	b
UC4082	*Pseudomonas pseudoalcaligenes*	+	Level 1	0.50 ± 0.05	b	0.84 ± 0.09	b
UC4083	*Stenotrophomonas acidaminiphila*	−	−	1.61 ± 1.35	ab	1.45 ± 1.11	b
UC4084	*Kosakonia radicincintans*	+	Level 2	1.20 ± 0.91	ab	0.50 ± 0.25	b
UC4086	*Klebsiella oxytoca*	−	Level 2	17.84 ± 0.94	ab	10.30 ± 0.55	ab
UC4087	*Pseudomonas indoloxydans*	+	−	1.62 ± 0.97	ab	2.05 ± 0.26	b
UC4088	*Pseudomonas indoloxydans*	+	−	1.88 ± 0.71	ab	1.99 ± 0.20	b
UC4089	*Stenotrophomonas pictorum*	+	−	5.82 ± 5.01	ab	0.66 ± 0.43	b
UC4090	*Aeromonas caviae*	+	Level 2	5.82 ± 2.37	ab	3.99 ± 0.26	ab
UC4091	*Pseudomonas pseudoalcaligenes*	+	−	7.32 ± 5.71	ab	1.61 ± 0.46	b
UC4092	*Kosakonia radicincitans*	+	Level 2	4.20 ± 0.51	ab	4.17 ± 0.91	ab
UC4093	*Stenotrophomonas pictorum*	+	Level 1	0.50 ± 0.12	b	0.38 ± 0.25	b
UC4094	*Enterobacter tabaci*	+	Level 2	15.33 ± 11.40	ab	13.88 ± 11.54	ab
UC4096	*Stenotrophomonas pavanii*	+	Level 1	1.42 ± 1.04	ab	0.90 ± 0.34	b
UC4098	*Stenotrophomonas rhizophila*	+	Level 2	5.20 ± 1.72	ab	33.07 ± 29.25	a
UC4099	*Enterobacter tabaci*	+	Level 2	11.84 ± 7.00	ab	3.05 ± 0.03	ab
UC4101	*Klebsiella grimontii*	+	Level 2	8.14 ± 0.18	ab	8.20 ± 0.35	ab
UC4102	*Chryseobacterium ureilyticum*	−	Level 1	1.23 ± 1.23	ab	1.13 ± 0.17	b
UC4103	*[Pseudomonas] hibiscicola*	+	Level 1	1.24 ± 1.23	ab	1.16 ± 0.51	b
UC4104	*Stenotrophomonas rhizophila*	+	Level 1	1.25 ± 1.15	ab	0.81 ± 0.37	b
UC4105	*Stenotrophomonas pictorum*	+	Level 1	5.65 ± 5.51	ab	0.93 ± 0.15	b
UC4106	*Enterobacter ludwigii*	+	Level 2	11.95 ± 3.33	ab	8.51 ± 0.91	ab
UC4107	*Sphingobacterium canadense*	−	−	1.90 ± 1.77	ab	0.67 ± 0.26	b
UC4108	*Chryseobacterium rhizosphaerae*	−	−	1.22 ± 0.90	ab	0.90 ± 0.18	b
UC4109	*Enterobacter tabaci*	+	Level 2	33.00 ± 27.14	a	3.71 ± 1.12	ab
UC4110	*Kosakonia oryzendophytica*	+	Level 2	2.05 ± 1.12	ab	1.27 ± 0.83	b
UC4112	*Pseudomonas taiwanensis*	+	Level 2	0.44 ± 0.06	b	1.02 ± 0.12	b
UC4113	*[Pseudomonas] hibiscicola*	+	Level 1	1.41 ± 1.37	ab	1.05 ± 0.61	b
UC4117	*Pseudomonas taiwanensis*	+	Level 2 (+)	2.21 ± 1.18	ab	2.90 ± 2.37	ab
UC4118	*Klebsiella oxytoca*	+	Level 2	8.55 ± 1.11	ab	7.37 ± 0.75	ab
UC4120	*Chryseobacterium rhizosphaerae*	−	−	5.80 ± 5.70	ab	0.72 ± 0.10	b
UC4121	*Sphingobacterium siyangense*	−	−	0.03 ± 0.03	b	0.05 ± 0.05	b
UC4122	*Pseudomonas taiwanensis*	+	Level 2 (+)	0.00 ± 0.00	b	1.00 ± 0.00	b
UC4123	*Klebsiella oxytoca*	+	Level 2	8.35 ± 1.71	ab	7.11 ± 1.04	ab
UC4125	*Delftia tsuruhatensis*	+	−	0.17 ± 0.10	b	0.49 ± 0.20	b
UC4126	*Pseudomonas japonica*	+	Level 2	5.94 ± 5.38	ab	4.30 ± 3.78	ab
UC4127	*Klebsiella oxytoca*	+	Level 2	8.38 ± 1.21	ab	7.12 ± 0.72	ab

The symbol + represents the presence of growth, while symbol − represents the absence of growth. S.e.m is indicative for Standard Error of the Mean. Try is indicative for DL-Tryptophan. Values are the Mean ± SE. Data for *N* fixation and *P* solubilization are the mean values of three replicates for each isolate. Data for IAA production are the mean values of two replicates for each isolate. Tukey-test: Values with different combinations of letters are significantly different from each other (*p* < 0.05).

**Table 3:** Biocontrol traits of the rhizobacteria.

Code	Identity	Antifungal Activity vs. *Sclerotinia sclerotiorum*		Siderophore	
		s.e.m	Tukey	s.e.m	Tukey
UC4080	*Sphingobacterium detergens*	21.40 ± 10.02	ab	41.50 ± 4.04	abcdef
UC4081	*Chryseobacterium oranimense*	31.77 ± 10.45	ab	47.66 ± 4.94	abcd
UC4082	*Pseudomonas pseudoalcaligenes*	43.18 ± 2.45	ab	31.40 ± 3.11	bcdefghi
UC4083	*Stenotrophomonas acidaminiphila*	37.00 ± 7.74	ab	42.91 ± 1.48	abcde
UC4084	*Kosakonia radicincintans*	31.03 ± 2.83	ab	30.38 ± 4.42	cdefghij
UC4086	*Klebsiella oxytoca*	20.00 ± 11.55	ab	10.08 ± 3.55	jklm
UC4087	*Pseudomonas indoloxydans*	39.20 ± 4.39	ab	28.80 ± 1.25	defghijkl
UC4088	*Pseudomonas indoloxydans*	41.08 ± 2.81	ab	27.58 ± 0.78	defghijkl
UC4089	*Stenotrophomonas pictorum*	39.08 ± 5.44	ab	51.36 ± 3.76	ab
UC4090	*Aeromonas caviae*	35.31 ± 13.80	ab	15.91 ± 7.60	hijklm
UC4091	*Pseudomonas pseudoalcaligenes*	44.19 ± 2.48	ab	29.50 ± 2.57	defghijk
UC4092	*Kosakonia radicincitans*	31.79 ± 6.41	ab	3.21 ± 2.00	mn
UC4093	*Stenotrophomonas pictorum*	28.92 ± 13.80	ab	47.73 ± 2.16	abcd
UC4094	*Enterobacter tabaci*	24.53 ± 4.55	ab	26.86 ± 1.27	efghijkl
UC4096	*Stenotrophomonas pavanii*	36.49 ± 8.46	ab	40.17 ± 1.08	abcdefg
UC4098	*Stenotrophomonas rhizophila*	27.24 ± 9.31	ab	0.00 ± 0.00	n
UC4099	*Enterobacter tabaci*	21.38 ± 6.48	ab	26.51 ± 2.84	efghijkl
UC4101	*Klebsiella grimontii*	29.19 ± 9.22	ab	0.56 ± 0.56	n
UC4102	*Chryseobacterium ureilyticum*	35.46 ± 3.22	ab	51.20 ± 3.11	ab
UC4103	*[Pseudomonas] hibiscicola*	45.10 ± 9.59	ab	35.41 ± 0.83	abcdefgh
UC4104	*Stenotrophomonas rhizophila*	26.96 ± 9.46	ab	30.51 ± 2.80	cdefghij
UC4105	*Stenotrophomonas pictorum*	35.98 ± 8.49	ab	40.90 ± 2.22	abcdef
UC4106	*Enterobacter ludwigii*	29.03 ± 5.49	ab	3.95 ± 2.36	mn
UC4107	*Sphingobacterium canadense*	22.12 ± 8.13	ab	52.28 ± 2.65	a
UC4108	*Chryseobacterium rhizosphaerae*	31.40 ± 0.36	ab	42.59 ± 8.01	abcde
UC4109	*Enterobacter tabaci*	18.07 ± 6.69	ab	8.79 ± 3.65	lmn
UC4110	*Kosakonia oryzendophytica*	29.02 ± 9.36	ab	9.64 ± 4.47	klm
UC4112	*Pseudomonas taiwanensis*	8.09 ± 0.26	ab	12.25 ± 1.14	ijklm
UC4113	*[Pseudomonas] hibiscicola*	40.85 ± 7.09	ab	35.53 ± 1.69	abcdefgh
UC4117	*Pseudomonas taiwanensis*	28.17 ± 5.93	ab	20.29 ± 8.28	ghijklm
UC4118	*Klebsiella oxytoca*	20.59 ± 6.29	ab	5.86 ± 4.65	mn
UC4120	*Chryseobacterium rhizosphaerae*	39.36 ± 8.38	ab	50.69 ± 1.16	abc
UC4121	*Sphingobacterium siyangense*	19.02 ± 6.57	ab	45.71 ± 5.14	abcde
UC4122	*Pseudomonas taiwanensis*	9.73 ± 3.34	ab	11.30 ± 2.24	ijklm
UC4123	*Klebsiella oxytoca*	44.56 ± 5.46	ab	1.24 ± 1.24	n
UC4125	*Delftia tsuruhatensis*	42.31 ± 3.95	ab	14.39 ± 1.44	ijklm
UC4126	*Pseudomonas japonica*	5.23 ± 2.62	b	21.77 ± 5.78	fghijklm
UC4127	*Klebsiella oxytoca*	48.01 ± 1.97	a	4.93 ± 3.18	mn

s.e.m indicates Standard Error of the Mean.

**Table 4:** Ranking of the rhizobacteria based on their in vitro PGP (plant growth promoting) and antifungal assay.

Code	Identity	*N* Fixation	*P* Solubilization	IAA Production		Antifungal Activity vs. *S. sclerotiorum*	Siderophore	Rank
				w/Try	w/o Try			
UC4094	*Enterobacter tabaci*	1	0.5	0.46	0.42	0.51	0.51	3.41
UC4098	*Stenotrophomonas rhizophila*	1	0.5	0.16	1,00	0.57	0.00	3.22
UC4109	*Enterobacter tabaci*	1	0.5	1.00	0.11	0.38	0.17	3.16
UC4127	*Klebsiella oxytoca*	1	0.5	0.25	0.22	1.00	0.09	3.06
UC4089	*Stenotrophomonas pictorum*	1	0.5	0.25	0.22	1.00	0.09	2.99
UC4105	*Stenotrophomonas pictorum*	1	0.25	0.17	0.03	0.75	0.78	2.98
UC4103	*[Pseudomonas] hibiscicola*	1	0.25	0.04	0.04	0.94	0.68	2.94
UC4123	*Klebsiella oxytoca*	1	0.5	0.25	0.21	0.93	0.02	2.92
UC4099	*Enterobacter tabaci*	1	0.5	0.36	0.09	0.45	0.51	2.90
UC4117	*Pseudomonas taiwanensis*	1	0.75	0.07	0.09	0.59	0.39	2.88
UC4113	*[Pseudomonas] hibiscicola*	1	0.25	0.04	0.03	0.85	0.68	2.86
UC4096	*Stenotrophomonas pavanii*	1	0.25	0.04	0.03	0.76	0.77	2.85
UC4090	*Aeromonas caviae*	1	0.5	0.18	0.12	0.74	0.30	2.84
UC4106	*Enterobacter ludwigii*	1	0.5	0.36	0.26	0.60	0.08	2.80
UC4093	*Stenotrophomonas pictorum*	1	0.25	0.02	0.01	0.60	0.91	2.79
UC4082	*Pseudomonas pseudoalcaligenes*	1	0.25	0.01	0.03	0.90	0.60	2.79
UC4084	*Kosakonia radicincitans*	1	0.5	0.04	0.02	0.65	0.58	2.78
UC4091	*Pseudomonas pseudoalcaligenes*	1	0	0.22	0.05	0.92	0.57	2.76
UC4101	*Klebsiella grimontii*	1	0.5	0.25	0.25	0.61	0.01	2.61
UC4118	*Klebsiella oxytoca*	1	0.5	0.26	0.22	0.43	0.11	2.52
UC4088	*Pseudomonas indoloxydans*	1	0	0.06	0.06	0.86	0.53	2.50
UC4087	*Pseudomonas indoloxydans*	1	0	0.05	0.06	0.82	0.55	2.48
UC4092	*Kosakonia radicincitans*	1	0.5	0.13	0.13	0.66	0.06	2.48
UC4104	*Stenotrophomonas rhizophila*	1	0.25	0.04	0.02	0.56	0.58	2.46
UC4110	*Kosakonia oryzendophytica*	1	0.5	0.06	0.04	0.60	0.18	2.39
UC4126	*Pseudomonas japonica*	1	0.5	0.18	0.13	0.11	0.42	2.33
UC4122	*Pseudomonas taiwanensis*	1	0.75	0.00	0.03	0.20	0.22	2.20
UC4125	*Delftia tsuruhatensis*	1	0	0.01	0.01	0.75	0.28	2.05
UC4102	*Chryseobacterium ureilyticum*	0	0.25	0.04	0.03	0.74	0.98	2.04
UC4120	*Chryseobacterium rhizosphaerae*	0	0	0.18	0.02	0.82	0.97	1.99
UC4086	*Klebsiella oxytoca*	0	0.5	0.54	0.31	0.42	0.19	1.96
UC4112	*Pseudomonas taiwanensis*	1	0.5	0.01	0.03	0.17	0.23	1.95
UC4081	*Chryseobacterium oranimense*	0	0.25	0.04	0.03	0.66	0.91	1.89
UC4083	*Stenotrophomonas acidamiphila*	0	0	0.05	0.04	0.77	0.75	1.61
UC4107	*Sphingobacterium canadense*	0	0	0.06	0.02	0.46	1.00	1.54
UC4108	*Chryseobacterium rhizosphaerae*	0	0	0.04	0.03	0.65	0.81	1.53
UC4080	*Sphingobacterium detergens*	0	0	0.04	0.01	0.45	0.79	1.29
UC4121	*Sphingobacterium siyangense*	0	0	0.00	0.00	0.40	0.87	1.27

w/Try and w/o Try stands for with or without DL-Tryptophan.
